# Work-related musculoskeletal complaints: risk factors and impact on work productivity among university administrative employees

**DOI:** 10.1186/s42506-024-00156-w

**Published:** 2024-05-15

**Authors:** Bassma A. Ibrahim, Samar E. M. Gaafar

**Affiliations:** Department of Public Health, Community Medicine, Environmental Medicine, and Occupational Medicine, Faculty of Medicine, Suez Canal University, Ismailia, Egypt

**Keywords:** Work-related musculoskeletal complaints, Administrative employees, Risk factors, Nordic Musculoskeletal Questionnaire, World Health Organization Health and Work Performance Questionnaire (HPQ)

## Abstract

**Background:**

Work-related musculoskeletal disorders (WMSDs) are a significant workplace problem leading to loss of productivity and disability. Administrative workers perform computer-based tasks for long periods. Consequently, they are at risk of developing musculoskeletal disorders. The objective of this study was to explore the frequency and risk factors of work-related musculoskeletal complaints and their impact on work productivity among administrative employees of Suez Canal University, Egypt.

**Methods:**

This cross-sectional study was conducted on 300 administrative employees through simple random sampling. Data were collected by an interview questionnaire including sociodemographic, work-related data, ergonomic and psychological risk factors, the Nordic Musculoskeletal Questionnaire (NMQ), and the World Health Organization Health and Work Performance Questionnaire (HPQ).

**Results:**

The frequency of work-related musculoskeletal complaints in at least one anatomical region over the past year was 74.7%. Neck (47.1%), lower back (40.7%), and shoulder (36.3%) were the most reported sites of complaints. Risk factors significantly associated with work-related musculoskeletal complaints were gender, age, physical activity, work experience, workplace stress, sustained body position, awkward posture, and inadequate rest breaks. Logistic regression revealed that older age (OR = 1.039, *p* = 0.023), being female (OR = 2.175, *p* = 0.011), and not having adequate rest breaks (OR = 1.979, *p* = 0.019) were significant predictors for the occurrence of WMSDs. The risk factors of absenteeism include gender, age, marital status, educational level, physical activity, BMI, work experience, and musculoskeletal complaints.

**Conclusion:**

Musculoskeletal problems were highly prevalent among administrative employees. Being female and not having adequate rest breaks were significant predictors for the occurrence of WMSDs. Ergonomic interventions and improvement of working conditions are recommended to reduce WMSDs.

## Introduction

Musculoskeletal disorders (MSDs) represent a prevalent occupational challenge that may lead to significant impacts, including physical disorders, disability, and a considerable economic strain on societies. Consequently, they are regarded as a prominent public health concern in both developed and developing nations [1].

MSDs denote inflammation and degeneration of the locomotor system, leading to pain, discomfort, and limited work and social involvement [2]. Administrative staff spends more time performing computer-based tasks requiring prolonged periods of sitting. Their work is characterized by a fast-paced environment, static and uncomfortable postures, repetitive movements, and unsuitable workplace conditions. These factors increase the risk of developing musculoskeletal disorders (MSDs), adversely affecting the well-being and welfare of employees and, consequently, diminishing overall productivity [3].

Employee productivity is a measure of how efficiently a worker performs. There are two methods for assessing the decrease in worker productivity. The initial approach is self-reported sickness absence (absenteeism), and the second method is presenteeism, which refers to the self-reported reduction in productivity while carrying out specific job tasks [4, 5].

An Iranian study conducted among office workers revealed that the most prevalent musculoskeletal issues during the preceding week were primarily associated with the lower back (41.6%), neck (41.6%), and shoulders (40.6%). Additionally, a positive correlation between the severity of discomfort experienced in the neck, lower back, and thighs and productivity was observed [6]. In a Malaysian study among office workers, there was a high prevalence (83.7%) of work-related musculoskeletal disorders (WMSDs) in various parts of the body. The most commonly reported symptom of MSDs was low back pain (58.5%). Similar to the Iranian study, this study found a statistically significant relationship between the prevalence of WMSDs and loss of productivity in the form of presenteeism [7].

The profound physical and economic effects of WMSDs emphasize the importance of addressing the problem among administrative employees. Furthermore, there is limited understanding of the relationship between WMSDs and work productivity. This study aims to determine the frequency of musculoskeletal complaints and their risk factors among administrative employees of Suez Canal University and to examine the association between musculoskeletal complaints and work productivity in terms of absenteeism and presenteeism.

## Methods

### Study design

This is a cross-sectional study conducted among administrative employees of Suez Canal University, Egypt, from January 2022 to August 2023.

### Study population and setting

The studied administrative employees were recruited from the administration of Personnel Affairs at Suez Canal University. Both male and female employees who had been employed for at least 1 year were enrolled in the study. The employees who had a history of musculoskeletal problems before their current employment or who had any disability that could affect their musculoskeletal health were excluded.

### Sample size and sampling technique

The sample size was calculated using EpiInfo StatCalc software, version 7.2.4.0. Assuming a prevalence of musculoskeletal problems among administrative employees to be 83.7% [7], a 5% margin of error, and a 95% confidence level, the calculated sample size was 211 participants, and we aimed to recruit 300 participants to account for potential non-response. Participants were recruited using simple random sampling. A list of 423 administrative employees from the administration of Personnel Affairs at Suez Canal University was obtained and we randomly selected individuals using a table of random numbers.

### Data collection

Data was collected by conducting face-to-face interviews using a structured questionnaire. The questionnaire was designed based on previous studies and a validated scale. It included sociodemographic data, work-related factors, ergonomic factors, psychosocial factors, and lifestyle behavior. The Nordic Musculoskeletal Questionnaire (NMQ) was employed to identify musculoskeletal discomfort in nine parts of the body, including the neck, shoulders, elbows, upper back, lower back, wrists/hands, hips/thighs/buttocks, knees, ankles, or feet in the previous 12 months and prior 7 days [8].

The World Health Organization Health and Work Performance Questionnaire (HPQ) was employed to evaluate work productivity among studied employees. In HPQ, the assessment of work productivity relies on measuring absenteeism and presenteeism in days through a series of self-administered questions [9]. A forward–backward translation approach was used to translate the English questionnaire. The questions were translated into Arabic by two language experts and then back-translated into English by two other experts. The questions were revised by two public health experts for face and content validity. Reliability assessment showed that Cronbach’s alpha was 0.8.

### Statistical analysis

The data collected from the study were analyzed using the statistical software SPSS version 25. Descriptive statistics were used for describing and summarizing data as appropriate (mean and standard deviation for continuous variables and frequency and percentage for categorical variables). We calculated the frequency of musculoskeletal complaints in at least one anatomical region. We reviewed the responses and determined whether each participant reported experiencing symptoms in any of the anatomical regions covered by the Nordic questionnaire. If a participant reported symptoms in at least one body region, we considered him as having an MSD in at least one anatomical region. When calculating the frequency in at least one anatomical region, each participant is counted only once, regardless of the number of regions they reported symptoms. This approach avoids double-counting individuals with symptoms in multiple regions, providing a measure of overall prevalence. Also, we calculated the prevalence of musculoskeletal complaints in a specific body region such as the neck, shoulders, arms, hands, back, and legs. The chi-square test was used to assess the relationship between the percentage of musculoskeletal problems and employees’ characteristics. The normality of data was tested with the Kolmogorov–Smirnov test of normality. Mann–Whitney *U* test was used to assess the association between participants’ characteristics, absenteeism, and presenteeism. Spearman’s correlation coefficient was conducted to evaluate the relationship between the frequency of musculoskeletal complaints and the ergonomic and psychological risk factors. Logistic regression analysis was performed to determine the independent predictors of musculoskeletal complaints. Statistical significance was considered if *p* < 0.05.

## Results

 There were 100 males (33.3%) and 200 females (66.7%). Their ages ranged from 22 to 59 years, with a mean age of 44.64 ± 8.73 years. Most of them were married (82.3%) and 57.3% had a high level of education. Forty-three (14.3%) of the participants were smokers, while 47 (15.7%) engaged in regular physical activity. The participant’s body mass index (BMI) values ranged from 20.02 to 49.95 kg/m^2^, with a mean of 29.26 ± 5.32 kg/m^2^. Seventy-three (24.3%) of the participants had a normal BMI, falling within the range of 18.5 to 24.9 kg/m^2^, while 113 (37.7%) were classified as obese, with a BMI greater than 28 kg/m^2^ as displayed in Table 1.

**Table 1:** Sociodemographic characteristics and lifestyle behavior of the studied Suez Canal University employees, 2022–2023 (*n* = 300)

Variables	No. (%)
Age (years) Mean ± SD	44.64 ± 8.73 (range: 22–59)
20-	13 (4.3)
30-	73 (24.3)
40-	111 (37.0)
50–60	103 (34.3)
Gender	
Male	100 (33.3)
Female	200 (66.7)
Marital status	
Married	247 (82.3)
Single	23 (7.7)
Divorced	18 (6.0)
Widow	12 (4.0)
Educational level	
Basic education	11 (3.7)
Secondary education	117 (39.0)
Higher education	172 (57.3)
Smoking	
Smoker	43 (14.3)
Non-smoker	257 (85.7)
Physically active	
Yes	47 (15.7)
No	253 (84.3)
Body mass index (BMI) Mean ± SD	29.26 ± 5.32
Normal	73 (24.3)
Overweight	114 (38.0)
Obese	113 (37.7)

The mean duration of work was 20.23 ± 8.90 years, with a range of 1 to 40 years. Among the participants, 39.3% had worked for 20–30 years. The mean absenteeism was 28.13 ± 24.70 hours/month. The mean presenteeism was 81.47 ± 19.51 hours/month (Table 2).

**Table 2:** Occupational characteristics of the studied Suez Canal University employees, 2022–2023 (*n* = 300)

**Variables**	**No. (%)**
Working experience (years) Mean ± SD	20.23 ± 8.90 (1–40)
< 10	33 (11.0)
10-	97 (32.3)
20-	118 (39.3)
30–40	52 (17.3)
Work productivity	
Absenteeism (hours/month) Mean ± SD	28.13 ± 24.70
Presenteeism (hours/month) Mean ± SD	81.47 ± 19.51

**Fig. 1:**
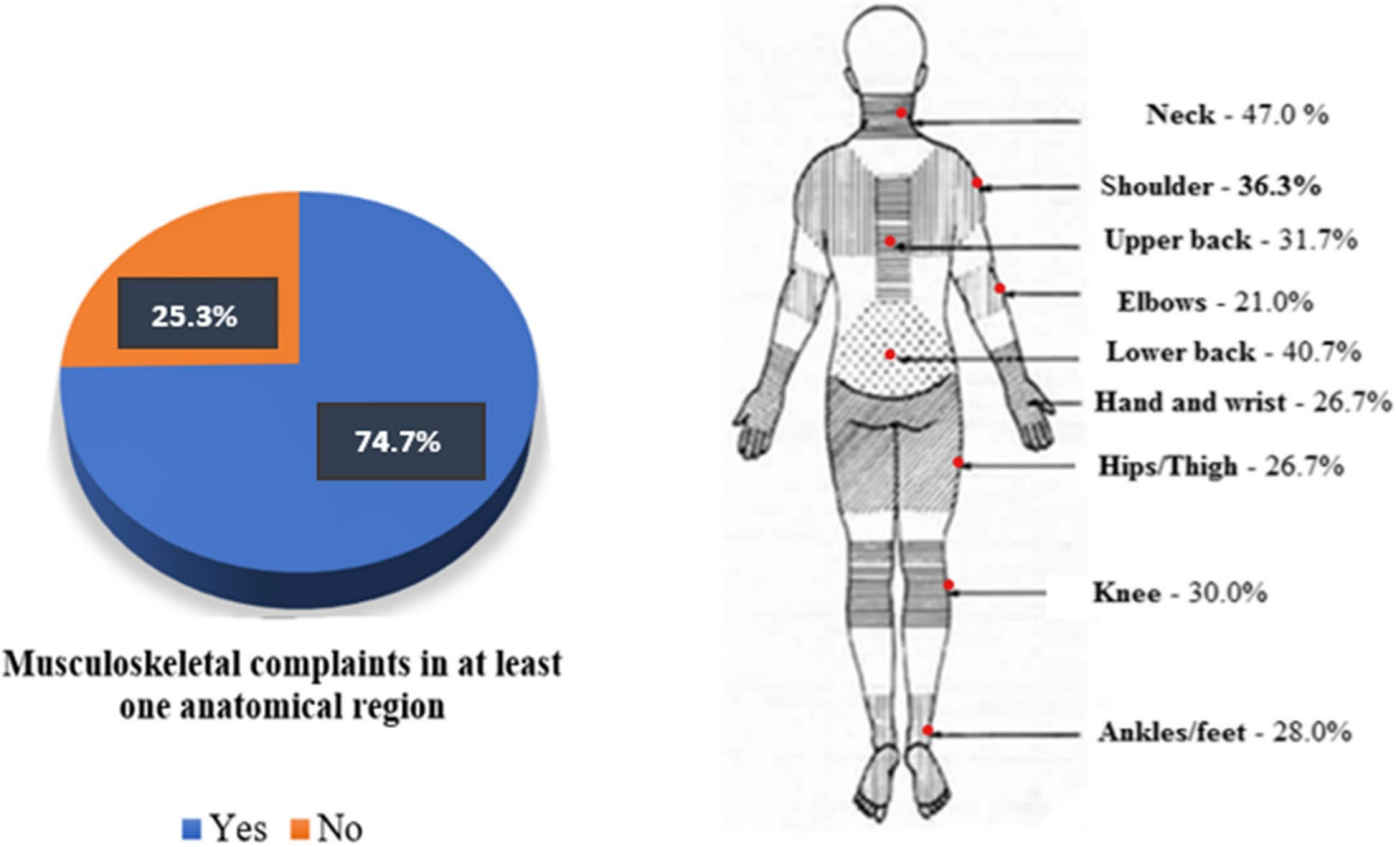
Frequency and sites of musculoskeletal complaints among studied employees (*n* = 300)

Out of the 300 participants, 224 (74.7%) experienced musculoskeletal complaints in at least one anatomical region over the preceding 12-month period. The neck (47.1%) was the most commonly affected anatomical region, followed by the lower back (40.7%). The elbows were the least frequently indicated anatomical regions, accounting for 21.0% of reported cases (Fig. 1).

As regards the relationship between employees’ characteristics and the occurrence of musculoskeletal complaints, being a female has a significant association with the prevalence of musculoskeletal complaints. Advancing age, increasing work experience, and physical activity also have a significant association with the prevalence of musculoskeletal complaints (Table 3).

**Table 3:** Association between the musculoskeletal problems and the employees’ characteristics (*n* = 300)

Characteristics	WMSDs		*p* value
	**Present** **No. (%)**	**Absent** **No. (%)**	
Gender			0.006*
Male	65 (65.0)	35 (35.0)	
Female	159 (79.5)	41 (20.5)	
Age (years)			0.021*
20-	7 (53.8)	6 (46.2)	
30-	47 (64.4)	26 (35.6)	
40-	89 (80.2)	22 (19.8)	
50–60	81 (78.6)	22 (21.4)	
Marital status			0.453^f^
Married	187 (75.7)	60 (24.3)	
Single	14 (60.9)	9 (39.1)	
Divorced	14 (77.8)	4 (22.2)	
Widower	9 (75.0)	3 (25.0)	
Educational level			0.100
Basic	7 (63.6)	4 (36.4)	
Secondary	95 (81.2)	22 (18.8)	
University	122 (70.9)	50 (29.1)	
Smoking			
Smoker	33 (76.7)	10 (23.3)	0.735
Non-smoker	191 (74.3)	66 (25.7)	
Physically active			
Yes	29 (61.7)	18 (38.3)	0.026*
No	195 (77.1)	58 (22.9)	
BMI			
Normal	51 (69.9)	22 (30.1)	
Overweight	84 (73.7)	30 (26.3)	0.377
Obese	89 (78.80)	24 (21.2)	
Working experience (years)			
1-	20 (60.6)	13 (39.4)	0.048*
10-	68 (70.1)	29 (29.9)	
20-	92 (78.0)	26 (22.0)	
30–40	44 (84.6)	8 (15.4)	

^f^Fisher exact test

^*^Statistically significant at *p* value < 0.05

Table 4 shows the results of the correlation analysis. There was a significant positive correlation between the prevalence of musculoskeletal complaints and the following reported risk factors: workplace stress, sustained body position, awkward posture, and inadequate rest breaks.

**Table 4:** Correlation between the musculoskeletal complaints and the ergonomic and psychological risk factors (*n* = 300)

Risk factor	*r*	*p* value
Workplace stress	0.120	0.037*
Multitasking	0.10	0.860
Not enough time for assignments	0.045	0.436
Cannot ask about anything concerning work	0.077	0.181
Find job assignments difficult	0.091	0.117
Do not get support from coworkers when making a mistake	0.031	0.588
Do not get support from supervisors when making a mistake	0.048	0.410
Sustained body position	0.184	0.001*
Awkward posture	0.130	0.025*
Improper bending	0.112	0.053
Repetitive movements	0.068	0.243
Work is physically exhausting	0.079	0.171
Inadequate rest break	0.129	0.026*

^*^Statistically significant at *p* value < 0.05

Moreover, the risk factors of absenteeism include gender, age, marital status, educational level, physical activity, BMI, work experience, and musculoskeletal complaints. However, there was no statistically significant association between presenteeism and the participants’ details (Table 5).

**Table 5:** Association between absenteeism and presenteeism and the participants’ characteristics (*n* = 300)

Characteristics	Absenteeism (hours per month) Mean ± SD	*p* value	Presenteeism (hours per month) Mean ± SD	*p* value
Gender				0.799
Male	23.24 ± 24.52	0.004*	81.90 ± 18.94	
Female	30.57 ± 24.48		81.25 ± 19.82	
Age (years)				0.374
20-	14.00 ± 19.06		89.23 ± 12.56	
30-	20.38 ± 18.19	0.001*	79.73 ± 20.21	
40-	27.53 ± 21.63		81.26 ± 20.19	
50–60	36.05 ± 29.58		81. 94 ± 1.87	
Marital status				0.052
Married	27.46 ± 23.73		81.91 ± 19.78	
Single	18.41 ± 18.07	0.028*	77.83 ± 19.06	
Divorced	36.17 ± 33.71		86.67 ± 18.75	
Widower	44.33 ± 31.03		76.67 ± 16.45	
Educational level				0.468
Basic	16.55 ± 14.43	0.035*	87.27 ± 11.03	
Secondary	32.34 ± 26.17		81.97 ± 20.39	
University	26.01 ± 23.76		80.76 ± 19.3	
Smoking				
Smoker	30.36 ± 29.43	0.844	79.53 ± 21.04	0.511
Non-smoker	27.75 ± 23.86		81.79 ± 19.26	
Physically active				
Yes	22.64 ± 24.91	0.039*	81.91 ± 19.41	0.799
No	29.15 ± 24.58		81.39 ± 19.56	
BMI				
Normal	23.21 ± 23.50		80.55 ± 17.95	
Overweight	25.97 ± 21.73	0.020*	81.14 ± 20.94	0.623
Obese	33.48 ± 27.34		82.39 ± 19.10	
Working experience (years)				
1-	19.93 ± 19.42		83.03 ± 20.23	
10-	24.10 ± 20.82	0.002*	79.70 ± 21.04	0.672
20-	27.14 ± 22.23		82.20 ± 18.82	
30–40	43.08 ± 32.96		82.12 ± 17.86	
Having MSDS				
Present	35.98 ± 23.57	0.000*	80.22 ± 20.67	0.106
Absent	4.97 ± 7.24		85.13 ± 15.10	

^*^Statistically significant at *p* value < 0.05

A multiple binary logistic regression was carried out to assess the effect of age, gender, physical activity, workplace stress, awkward posture, sustained position, and rest breaks on the likelihood of musculoskeletal complaints. The overall model was statistically significant when compared to the null model, explaining 18.2% of the variation of musculoskeletal complaints (Nagelkerke *R*^2^ = 0.182). Older age, being female, and not having adequate rest breaks were significant predictors for the occurrence of musculoskeletal complaints among our study participants (Table 6).

**Table 6:** Regression analysis for the predictors of musculoskeletal complaints among the university employees (*n* = 300)

Predictor	*p* value	OR	95% CI
Age	0.023*	1.039	1.039–1.005
Gender			
Male (ref.)		1	
Female	0.011*	2.175	1.196–3.957
Physically active			
Yes	0.187	0.614	0.297–1.267
No (ref.)		1	
Workplace stress	0.242		
Never (ref.)		1	
Sometimes	0.145	1.838	0.810–4.168
Always	0.114	1.851	0.862–3.974
Sustained position	0.132		
Never (ref.)		1	
Sometimes	0.458	1.393	0.580–3.343
Always	0.059	2.165	0.971–4.828
Awkward posture	0.276		
Never (ref.)		1	
Sometimes	0.110	1.987	0.857–4.608
Always	0.299	1.481	0.706–3.106
Having rest break			
Yes (ref.)		1	
No	0.019*	1.979	1.116–3.507
Constant	0.002	0.064	

*OR*, odds ratio; *CI*, confidence interval

^*^Statistically significant *p* value (< 0.05)

## Discussion

This study revealed a high frequency of musculoskeletal complaints (74.7%) among administrative employees at least in one region of the body during the previous 12 months. The most common body region reported by the present cohort was the neck (47.1%), followed by the lower back (40.7%) and shoulder (36.3%).

This could be attributed to the inadequate ergonomic knowledge and practices employed by the administrative personnel, with the application of a continuous work pattern over extended periods without adequate breaks, the maintenance of static and awkward postures, and workplace environments with poor ergonomic circumstances.

Similarly, a Nigerian study among office workers in higher education institutions demonstrated a high prevalence rate of musculoskeletal complaints (71.9%). It also reported that body regions affected by musculoskeletal complaints were the lower back (58.1%) and shoulders (50.2%) [10]. Similar results were reported in studies in Iran, Turkey, and Jordan which indicated that the prevalence rates of neck, shoulders, and lower back symptoms were higher among administrative employees [11–13].

However, a Saudi study revealed a much higher prevalence of musculoskeletal problems (84.5%) for Saudi office workers in the past 12 months [14] and it also reported a similar pattern of the most affected areas in the form of low back region (54.5%), shoulder (51.7%), and neck (50.1%). These variations could be attributed to the sociocultural differences and the differences in the work setting.

Another important finding was that general and occupational characteristics including gender, age, duration of employment, and physical activity were statistically associated with developing musculoskeletal complaints. The rate of musculoskeletal complaints was higher in females, those over the age of 40 years, those with work experience higher than 10 years, and those who were physically inactive. Additionally, multiple regression revealed that older age (OR = 1.039) and female gender (OR = 2.175) were predictors for WMSDs. Regarding gender, similar previous studies have reported that female office workers were more affected by musculoskeletal complaints than men [10, 12, 15]. Women may experience varying anthropometrics and physiology which could explain the observed differences. Additionally, women frequently undertake non-work-related tasks, such as household chores, which may contribute to muscle strain and increase their susceptibility to WMSDs [16].

These results are in line with those of Ahmed and Oraby in Egypt who also found that age more than 40 years and duration of work more than 10 years were the most significant predictors of work-related musculoskeletal disorders [17]. Likewise, many studies have reported a significant association between the prevalence of WMSDs and both advancing age and increasing work experience [10, 14, 18]. The aging process can lead to degenerative changes and a decline in functional capacity among older workers. This, in turn, may enhance the body’s susceptibility to mechanical stress and make it more vulnerable to MSDs. Additionally, chronic musculoskeletal fatigue can result in the accumulation of stress on muscles and tendons, ultimately leading to reduced blood flow to the corresponding areas as work experience increases [19].

 Our findings regarding physical activity align with the results of studies from Turkey and Indonesia. These studies have shown a clear significant association between physical activity habits and the prevalence of MSDs. Additionally, the lack of exercise has been found to increase the occurrence of these problems [20, 21]. This could be attributed to the impact that physical activity has on the status of muscles, as it increases their mass and functionality. Therefore, the absence of physical activity, coupled with low body fitness, may result in reduced flexibility and an elevated risk of MSDs [19].

In terms of the factors that contribute to the development of MSDs, both psychological and ergonomic aspects were considered. Upon conducting a correlation analysis, it was found that workplace stress, sustained body position, awkward posture, and inadequate rest breaks all had a statistically significant positive correlation with the development of MSDs. Multivariate analysis further demonstrated that insufficient rest breaks is a significant predictor of MSDS (OR = 1.979).

This finding demonstrates similarities to a study conducted in Nigeria, which found that awkward posture, workplace stress, inadequate rest breaks, and sustained body position were significantly associated with MSDS [10]. Another study by Ahmed and Oraby also supports these results, revealing that awkward body postures play a significant role as a risk factor for the development of WMSD (OR = 2.46) [17]. Similarly, Celik et al. [12], as well as Ardahan and Simsek [11], reported that working without adequate rest breaks was a risk factor for WMSD. A possible reason could be that awkward positions often necessitate the body to assume unnatural and unbalanced positions. Consequently, this leads to an increase in muscle tension, a decrease in blood flow, and an increase in pressure on joints and nerves, ultimately resulting in microtrauma and inflammation in the affected tissues. The association between not taking sufficient rest breaks and musculoskeletal disorders may be linked to the continuous and excessive load placed on the muscles and joints. Without proper rest, the tissues are unable to recover and adapt, and the wear and tear on the musculoskeletal system increases over time.

Similar studies on workplace stress have found that work-related stress can increase the risk of musculoskeletal problems among employees [22, 23]. Occupational stress can have an impact on nerves, hormones, and blood pressure, which can result in increased muscle and skeletal activity. This can put extra strain on the musculoskeletal system, potentially leading to the development or worsening of MSDs.

Regarding work productivity, our results showed that the mean absenteeism was 28.13 ± 24.70 h/month, while the mean presenteeism was 81.47 ± 19.51 h/month. Furthermore, gender, age, marital status, educational level, physical activity, BMI, working experience, and development of WMSDS were significantly associated with absenteeism. The highest mean of absenteeism was found in females, those over the age of 50 years, widowers, those with secondary education, physically inactive individuals, obese, those with more than 30 years of work experience, and individuals with WMSDs.

Our study findings were similar to those of previous studies. A study by Dos Reis França et al. in Brazil, displayed an association between absenteeism and both advanced age and female gender [24]. Additionally, a prior study by Rodrigues et al. in Brazil found that absenteeism was significantly higher among females [25]. Our finding also aligns with the research conducted by Haeffner et al. in Brazil where they demonstrated a significant correlation between education and absenteeism [26]. Similar prior research found that absenteeism was associated with musculoskeletal complaints [27, 28].

Nevertheless, there was no statistically significant difference observed between presenteeism and employee characteristics, including the prevalence of musculoskeletal complaints, in this study. This finding is consistent with that of Balta and Alagüney in Turkey who did not find a relationship between musculoskeletal pain and presenteeism [29]. The finding is contrary to a previous study by Bae among physical therapists in Korea which found that WMSDs are associated with presenteeism and individuals exhibit significant presenteeism [15]. A potential explanation for this finding is that the influence of WMSDs on presenteeism could rely on different issues, like the seriousness of the condition, the nature of the job tasks, workplace culture, job satisfaction, and organizational support and how individuals cope with the studied population. Additionally, the subjective evaluation of occupational musculoskeletal exposures in the present study may also contribute to this explanation.

### Limitations of the study

This study has several limitations. Firstly, this study employs a cross-sectional design, which captures data at a specific point in time and therefore prevents the establishment of causal relationships between risk factors and musculoskeletal disorders. Longitudinal studies that track participants over time would provide a more comprehensive understanding of the prevalence and potential progression of musculoskeletal disorders among administrative employees. Secondly, this cross-sectional study was conducted in a specific university setting among the Egyptian administrative employee population. So, caution should be exercised when generalizing the findings beyond the study population. Thirdly, this study is based on self-reported data, which predisposes our results to recall bias.

## Conclusions

The present study found a high prevalence of musculoskeletal complaints among administrative employees (74.7%) with a high proportion experiencing symptoms in the neck, lower back, and shoulders. Additionally, we found that the significant risk factors for musculoskeletal complaints include female gender, increasing age, increased duration of employment, lack of physical activity, perception of workplace stress, sustained body position, awkward posture, and inadequate rest breaks. Moreover, regression analysis revealed that older age, being female, and not having enough rest breaks were significant predictors for the occurrence of musculoskeletal complaints. Also, there was a significant association between absenteeism and gender, age, marital status, educational level, physical activity, BMI, and working experience, as well as development of musculoskeletal complaints. However, the observed difference between presenteeism and employee characteristics including the frequency of musculoskeletal complaints in this study was not statistically significant.

Providing ergonomics training for administrative personnel is crucial to enhance their understanding of ergonomics, musculoskeletal disorders, the importance of taking regular rest breaks, and maintaining healthy postures. Furthermore, it is beneficial to motivate employees to engage in regular physical activity. Institutions are recommended to regularly monitor and make necessary adjustments to the workplace setting by offering adaptable workstations, ergonomic tools, and improved working conditions. Pre-employment and periodic medical examinations should be conducted to manage musculoskeletal complaints. Further research should be conducted on a larger scale to further assess the frequency and potential causes of musculoskeletal complaints among administrative employees.

## Data Availability

The datasets employed during the present investigation can be accessed from the corresponding author upon a reasonable request.

## Abbreviations

WMSDs: Work-related musculoskeletal disorders
HPQ: World Health Organization Health and Work Performance Questionnaire
SD: Standard deviation
